# *Moraxella catarrhalis *acquisition, airway inflammation and protease-antiprotease balance in chronic obstructive pulmonary disease

**DOI:** 10.1186/1471-2334-9-178

**Published:** 2009-11-15

**Authors:** Ganapathi I Parameswaran, Catherine T Wrona, Timothy F Murphy, Sanjay Sethi

**Affiliations:** 1 Division of Infectious Diseases, University at Buffalo, State University of New York, 701 Ellicott Street, Buffalo, NY 14203, USA; 2 Department of Microbiology, University at Buffalo, State University of New York, 701 Ellicott Street, Buffalo, NY 14203, USA; 3 Division of Pulmonary and Critical Care, University at Buffalo, State University of New York, 3495 Bailey Ave, Buffalo, NY 14215, USA; 4 VA Western New York Healthcare System, 3495 Bailey Ave, Buffalo, NY 14215, USA; 5 Kaleida Health System, 100 High Street, Buffalo, NY 14203, USA

## Abstract

**Background:**

*Moraxella catarrhalis *causes approximately 10% of exacerbations in chronic obstructive pulmonary disease (COPD) and also colonizes the lower airway in stable patients. Little is known about the effects of colonization by *M. catarrhalis *on airway inflammation and protease-antiprotease balance, and how these changes compare to those seen during exacerbations. Since COPD is a progressive inflammatory disease, elucidating the effects of bacterial colonization and exacerbation on airway inflammation is relevant to understanding disease progression in COPD. Our aims were (1) Analyze changes in airway inflammation in colonization and exacerbation of COPD due to *M. catarrhalis*; (2) Explore protease-antiprotease balance in colonization and exacerbation due to *M. catarrhalis*. Our hypothesis were (1) Acquisition of a new strain of M. catarrhalis in COPD increases airway inflammation from baseline and alters the protease-antiprotease balance towards a more proteolytic environment; (2) These changes are greater during exacerbations associated with *M. catarrhalis *as compared to colonization.

**Methods:**

Thirty-nine consecutive COPD patients with 76 acquisitions of a new strain of *M. catarrhalis *over a 6-year period were identified in a prospective study. Seventy-six pre-acquisition sputum supernatant samples, obtained just before acquisition of *M catarrhalis*, and 76 acquisition samples (34 were associated with exacerbation, 42 with colonization) were analyzed for IL-8, TNF-α, Neutrophil Elastase (NE) and Secretory leukocyte protease inhibitor (SLPI). Changes were compared in paired samples from each patient.

**Results:**

IL-8, TNF-α and NE were significantly elevated after acquisition of *M. catarrhalis*, compared to pre-acquisition samples (p =< 0.001 for all three). These changes were present in colonization (p = 0.015 for IL-8; p =< 0.001 for TNF-α and NE) as well as in exacerbation (p =< 0.001 for all three), compared to pre-acquisition levels. SLPI was significantly lower after acquisition (p =< 0.001), in colonization (p =< 0.001) as well as in exacerbation (p = 0.004), compared to pre-acquisition levels. SLPI levels correlated negatively with NE levels (R^2 ^= 0.07; p = 0.001).

**Conclusion:**

Acquisition of *M. catarrhalis *in COPD causes increased airway inflammation and worsening protease-antiprotease imbalance during exacerbations and also in colonization, even in the absence of increased symptoms. These effects could contribute to progression of airway disease in COPD.

## Background

Chronic obstructive pulmonary disease (COPD) is characterized by chronic airway inflammation, and progressive deterioration in lung function and general health status [1-4]. The course of COPD is punctuated by acute exacerbations, which are characterized by worsening respiratory symptoms, enhanced airway inflammation [5,6] and deterioration of lung function [7]. A variety of airway inflammatory mediators and cells increase with exacerbation, reflecting mainly neutrophilic and at times eosinophilic inflammation [4-8].

Approximately half of COPD exacerbations are caused by bacterial pathogens including nontypeable *Haemophilus influenzae, Moraxella catarrhalis, Streptococcus pneumoniae and Pseudomonas aeruginos*a [9-12]. These same pathogenic bacteria can be found in the lower airways of 30%-40% of COPD patients in the absence of symptoms of an acute exacerbation. This 'colonization' by potential pulmonary pathogens is also associated with increased airway inflammation; however these observations have been confined to cross-sectional studies [13-15]. During exacerbations of COPD, symptoms correlate with the level of airway inflammation [5,16,17]. Therefore, one would expect airway inflammation induced by colonization to be of lower intensity than that observed during an exacerbation. However, a direct comparison between airway inflammation associated with colonization and exacerbation in the same patient population has not been described.

Furthermore, previous studies of bacteria-associated inflammation in COPD, during colonization or exacerbation, have included several different bacterial pathogens. It is likely that the inflammatory response in the airways in COPD to different bacterial species is different. Studying the inflammatory response to a single pathogenic species is likely to be more consistent and allow one to better compare colonization with exacerbation.

An enduring hypothesis is that tissue damage in COPD is due to an imbalance between proteases, produced mostly by neutrophils and macrophages, and anti-proteases produced locally in the airways as well as transudating from plasma [18]. This imbalance is worsened during exacerbations of COPD [8,17]. Neutrophil elastase (NE) is the protease most implicated in causing tissue damage in COPD. SLPI is the main anti-protease in airway secretions [18,19]. The balance between NE and SLPI is altered in a detrimental way, tilting the balance toward proteolytic activity, during exacerbations [8]. Whether colonization by potential pulmonary pathogenic bacteria, without any change in baseline respiratory symptoms, can alter the protease-antiprotease balance in the airways is unknown.

We decided to study the effects of acquisition of pathogenic bacteria in COPD patients by individual bacterial species in order to minimize the variability in host response to different bacterial species. *M. catarrhalis *demonstrates well-defined periods of carriage in COPD and causes approximately 10% of acute exacerbations in COPD [12]. Therefore, we chose *M. catarrhalis *as the index pathogen in this study. Our hypothesis was that acquisition of a new strain of *M. catarrhalis *in COPD enhances airway inflammation from baseline and alters the protease-antiprotease balance towards a more proteolytic environment. We further hypothesize that these changes are greater during exacerbations associated with *M. catarrhalis *as compared to colonization by this pathogen.

## Methods

### COPD study clinic

Details of this study clinic have been described previously [5,11]. The Institutional Review Board of the VA WNY Healthcare system approved the study protocol. All participants gave written informed consent. Briefly, participants with smoking associated chronic bronchitis were enrolled and followed on a monthly basis and also whenever they suspected they were experiencing an exacerbation. At each clinic visit, clinical information, sputum and serum samples were collected. Patients were questioned about the status of their chronic respiratory symptoms (dyspnea, cough, sputum production, viscosity and purulence), and the responses were graded at 1 (at the usual level), 2 (somewhat worse than usual) or 3 (much worse than usual). A minor worsening of two or more symptoms or major worsening of one or more symptoms prompted clinical assessment of the cause. If the patient had fever (>38.3C), appeared ill or had signs of consolidation on examination of the lungs, a chest film was obtained to rule out pneumonia. If other causes of worsening of symptoms, such as pneumonia, upper respiratory tract infection, or congestive cardiac failure were ruled out, the patients was considered to be having an exacerbation of COPD. The determination of whether the patient had stable disease or an exacerbation was made before results of sputum cultures were available [5,11].

Acquisition was defined as the isolation of new strain of *M. catarrhalis *from sputum culture in a patient who did not have the same strain on sputum cultures previously. New strains were identified by pulsed field gel electrophoresis [12]. Colonization was defined as acquisition of a new strain of *M catarrhalis*, but with no change in baseline respiratory symptoms.

### Sputum Samples

Sputum samples were processed as described previously [5,11]. Briefly, spontaneously expectorated morning sputum samples were homogenized with the addition of dithiothreitol (Sputolysin, Calbiochem USA) and an aliquot processed for quantitative bacteriology. Sputum supernatants were obtained by centrifugation and stored at -80 degrees C.

During the period 1994-2000, a total of 50 patients acquired a new strain of *M catarrhalis *for a total of 120 acquisitions [12]. Of these, 57 (47.5%) acquisitions were associated with exacerbation, the rest 63 (52.5%) were colonization episodes in which no change in baseline symptoms occurred. Sputum samples obtained at the time of new strain acquisition (acquisition samples) as well as those obtained at a visit prior to the acquisition of *M. catarrhalis *(pre-acquisition samples), were identified. If an acquisition sample was not available, the corresponding pre-acquisition samples were also not included in this study. Pre-acquisition samples were obtained at a clinically stable state. Available samples closest to the acquisition sample were used.

### Sputum analysis

Sputum supernatant levels of interleukin-8 (IL-8), tumor necrosis factor (TNF-α) and active neutrophil elastase (NE) were measured as described earlier [5]. Secretory leukocyte protease inhibitor (SLPI) levels in sputum were measured by sandwich ELISA as follows. Ninety six-well plates (Corning, USA) were coated with 100 μl/well of monoclonal anti- human SLPI antibody (R&D Systems, Minneapolis, MN, USA) diluted to 2 μg/ml in 0.1 M sodium carbonate/0.1 M sodium bicarbonate buffer (pH 9.6) overnight at 4°C. Wells were blocked with 300 μl/well of 3% milk powder in PWB (1× Phosphate buffered saline with 0.05% Tween-20) for 1 hour at room temperature. Washing with PWB in between incubations, 100 μl volumes of standards (recombinant human SLPI, R&D Systems, Minneapolis, MN, USA) and samples (diluted 1:10,000 in 1% milk powder/PWB), were added to the wells for 37°C for 2 hours, followed by biotinylated anti-human SLPI antibody (R&D Systems, Minneapolis, MN, USA) diluted to 10 ng/ml in 1% milk powder/PWB, 100 μl/well for 1 hour at room temperature, followed by 100 μl/well of HRP labeled Streptavidin (Kirkegaard & Perry Laboratories, Gaithersburg, MD, USA) diluted 1:10,000 in 3% goat serum/PWB for 30 minutes at room temperature. Color development was done with Tetra-methyl benzidine (TMB) dissolved in Dimethyl sulfoxide (DMSO) at room temperature in dark for 15 minutes. The reaction was stopped by adding 100 μl/well of 1 M H_2_SO_4_. Absorbance at 450 nM was determined and SLPI levels in the sample wells were determined from the standard curve. The minimum concentration of SLPI detectable in this assay was 0.1 nanograms/ml and the linear range was 0.5-10 ng/ml.

### Statistical analysis

Data are presented as mean ± standard error (SEM) when normally distributed and as median ± interquartile range when non-normally distributed. Analyses were performed for paired data by comparing pre-acquisition and acquisition samples. Changes in log-transformed IL-8, TNF-α, NE and SLPI were compared with paired t-tests. Non-normally distributed paired data were analyzed with Wilcoxon paired rank sum tests. Comparisons between changes in colonization and exacerbation were done with Student 't' test. Correlation between NE and SLPI was assessed with linear regression. Statistical analyses were conducted using Analyse-it^® ^and Microsoft Excel^®^.

## Results

### Samples and Subjects

Out of a total of 120 *M. catarrhalis *new strain acquisitions during the period 1994-2000, 76 acquisition samples were available for analysis. The rest of the acquisition samples (44) had been used up in prior studies examining other aspects of *M. catarrhalis *in COPD. Each acquisition sputum sample, from which a new strain of *M. catarrhalis *was first isolated from a patient, was paired with a pre-acquisition sample from the same patient. A total of 76 such paired sputum samples from 39 patients were available for analysis, of which 34 (44.7%) acquisitions were associated with exacerbation, while 42 (55.3%) were colonization episodes. In the pre-acquisition samples, no potentially pathogenic bacteria were cultured from sputum. In the acquisition samples, *M. catarrhalis *was the only potentially pathogenic bacterial species found on sputum culture. There were 104 subjects who had at least one COPD study clinic visit during 1994-2000. Of these, 39 subjects contributed samples to this study and their demographics are shown in Table 1. The mean time between pre-acquisition and acquisition samples was 1.9 months (95% CI 1.6-2.3); median time between was 1 month (95% CI 1-2).

**Table 1:** Patient characteristics

Mean age in years (range)	65.9 (46-81)
Gender	Male:38; Female:1
Race	Caucasian:32; African-American:7
Mean years since diagnosis (range)	12.5(0-54)
Smoking status on enrollment	Current: 13; Ex-smokers:26
Mean smoking pack years (range)	76.3(10-175)
Mean FEV_1 _in liters (range)	1.62(0.47- 4.07)
Mean FEV_1_% predicted (range)	47.4(15-99)
GOLD severity (number in each group)	0 = 2; 1 = 1; 2 = 12; 3 = 18; 4 = 6

### Change in airways inflammation with *M. catarrhalis *acquisition

We measured sputum IL-8 and TNF-α to determine whether acquisition of *M catarrhalis *changes levels of airway inflammation. Baseline airway inflammation varies among patients with COPD. Therefore, all analyses were performed for paired data by comparing pre-acquisition and acquisition samples. In order to accurately measure the change in airway inflammation with acquisition, changes in log transformed values of the inflammatory markers were calculated by subtracting the pre-acquisition level from the acquisition level for each pair of samples (n = 76 pairs). Significant increase in IL-8 [2.63 ng/ml (2.11 ng/ml to 4.74 ng/ml) p < 0.0001] and TNF-α [0.05 ng/ml (0 ng/ml to 0.05 ng/ml) p < 0.0001] were seen with the acquisition of *M. catarrhalis *(Table 2).

**Table 2:** Levels of IL-8, TNF-α, NE and SLPI in sputum supernatants before and with acquisition of *M catarrhalis *(p values by Wilcoxon matched pairs rank test).

	Pre-acquisition Median (Interquartile range)	Acquisition Median (Interquartile range)	p value
**IL-8 ng/ml**	2.11 (5.09)	4.74 (17.5)	<0.001
**TNF-α ng/ml**	0 (0.02)	0.05 (0.15)	<0.001
			
**NE nM/ml**	0.12 (0.2)	8.42 (76.55)	<0.001
**SLPI μg/ml**	35.6 (47.75)	10.84 (24.19)	<0.001

**Figure 1:**
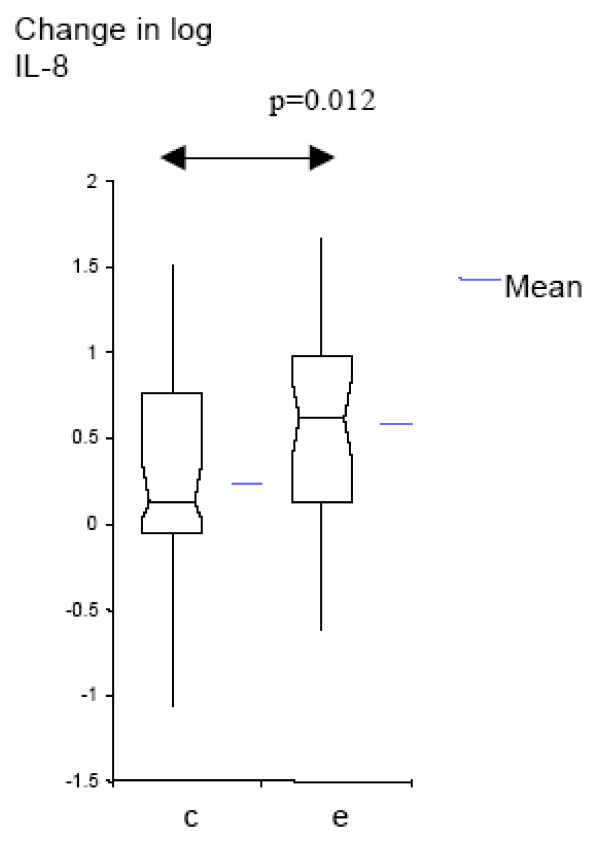
**Log_10 _change from IL-8 pre-acquisition levels for colonization and exacerbation samples (Box-plots showing means and 95% CI); c = colonization; e = exacerbation**.

**Figure 2:**
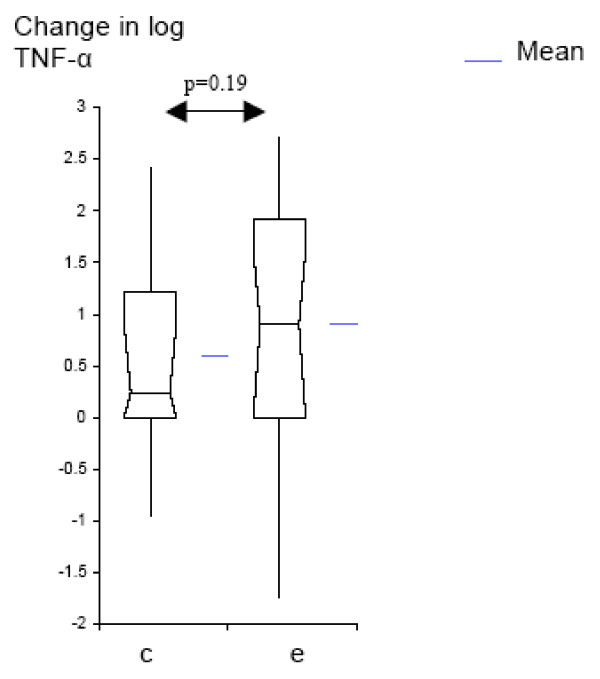
**Log_10 _change from TNF-α pre-acquisition levels for colonization and exacerbation samples (Box-plots showing means and 95% CI); c = colonization; e = exacerbation**.

We analyzed the data further to determine whether airway inflammation increases with colonization by *M. catarrhalis*, and to compare these changes with those seen with exacerbation. Colonization with *M. catarrhalis *was associated with significant increases from baseline in the levels of IL-8 (p = 0.015) and TNF-α (p =< 0.001). Exacerbations were associated with larger increases in airway inflammation than colonization (Table 3, Fig 1, 2). This increase from pre-acquisition levels was significantly larger in exacerbation, compared to the increase in colonization, for IL-8 (p = 0.012), but not for TNF-α (p = 0.197). We conclude that acquisition of a new strain of *M. catarrhalis *increases airway inflammation in COPD. This increase is seen with colonization by *M. catarrhalis *and to a larger extent during exacerbations.

**Table 3:** Changes in log_10 _levels of IL-8, TNF-α, NE and SLPI from pre-acquisition to acquisition, colonization and exacerbation samples. (p values by comparison to pre-acquisition levels by t test)

	**Acquisition-** **Mean change (95% CI)** **n = 76**	**Colonization samples only** **Mean change (95% CI)** **n = 34**	**Exacerbation samples only** **Mean change (95% CI)** **n = 42**
**Change in IL-8**	0.39 (0.25-0.53) (p =< 0.001)	0.23 (0.04-0.42) (p = 0.015)	0.59 (0.38-0.79) (p =< 0.001)
**Change in TNF-α**	0.72 (0.48-0.96) (p =< 0.001)	0.58 (0.31-0.85) (p =< 0.001)	0.89 (0.47-1.33) (p =< 0.001)
**Change in Elastase**	1.12 (0.79- 1.44) (p =< 0.001)	0.74 (0.32-1.16) (p =< 0.001)	1.58 (1.08-2.08) (p =< 0.001)
**Change in SLPI**	-0.51 (-0.72 to -0.30) (p =< 0.001)	-0.47 (-0.72- -0.23) (p =< 0.001)	-0.55 (-0.92- -0.19) (p = 0.004)

### Protease-antiprotease balance and *M. catarrhalis *acquisition

**Figure 3:**
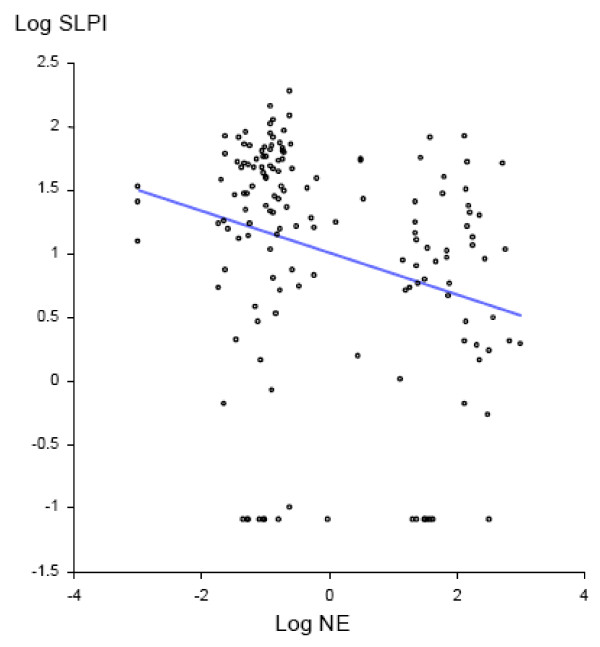
**Linear regression showing relationship of log-SLPIand log-NE levels for all samples (acquisition and pre-acquisition samples included) (R^2 ^= 0.07; p = 0.001)**.

SLPI is a major anti-protease in the airway lumen, while NE reflects uninhibited protease activity. We assessed changes in airway protease- antiprotease balance with acquisition of *M. catarrhalis*, by determining changes from baseline in NE and SLPI. With acquisition of *M. catarrhalis*, NE levels increased [8.3 nM/ml (0.12 nM to 8.42 nM), p < 0.0001] while SLPI levels decreased significantly [-24.76 μg/ml (35.6 μg to 10.84 μg) p =< 0.001] (Table 2). SLPI and NE levels had an inverse correlation by linear regression analysis (R^2 ^= 0.07; p = 0.001 for the slope) (Fig. 3).

**Figure 4:**
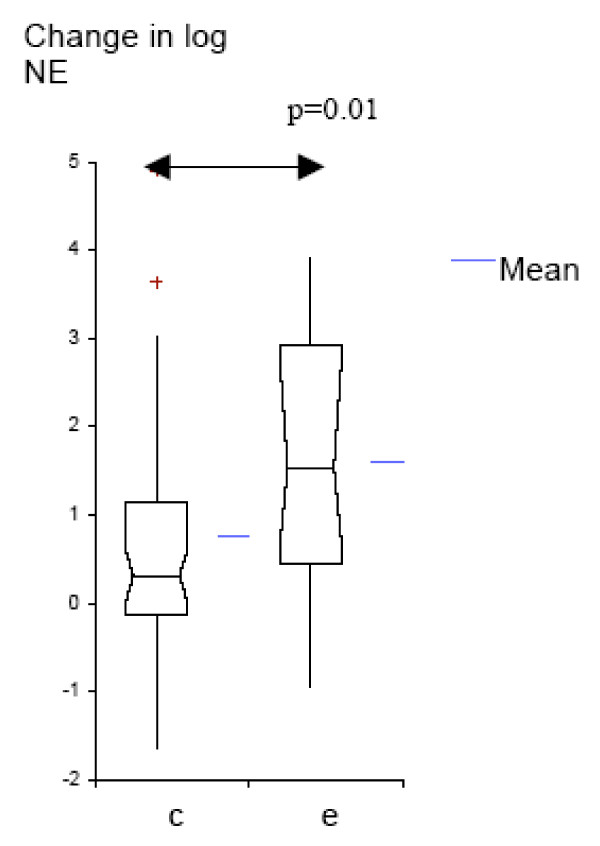
**Log_10 _change from NE pre-acquisition levels for colonization and exacerbation samples (Box-plots showing means and 95% CI); c = colonization; e = exacerbation**.

**Figure 5:**
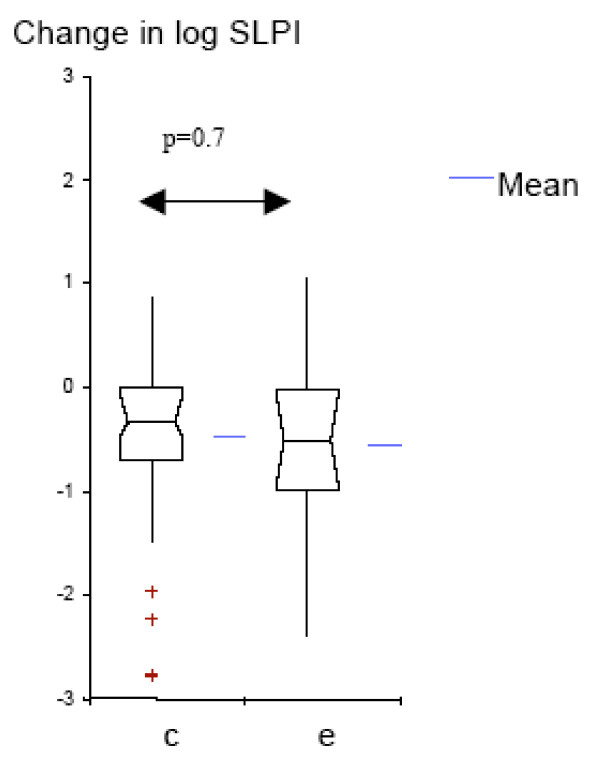
**Log_10 _change from SLPI pre-acquisition levels for colonization and exacerbation samples (Box-plots showing means and 95% CI); c = colonization; e = exacerbation**.

**Figure 6:**
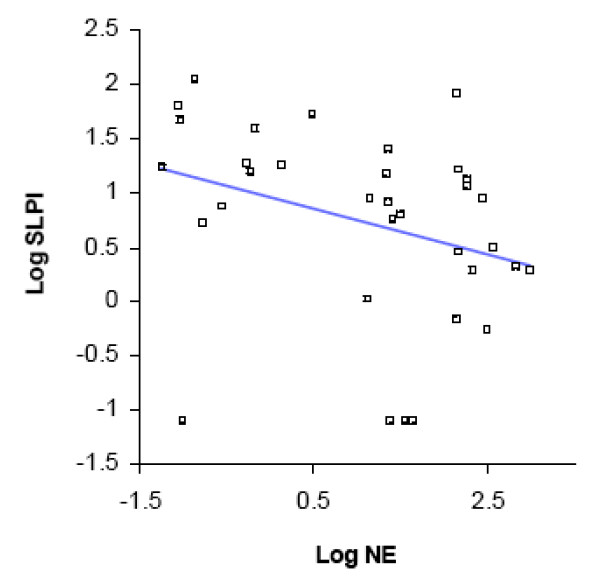
**Linear regression showing relationship of log-SLPI and log-NE levels for exacerbation samples only (R^2 ^= 0.1; p = 0.06)**.

**Figure 7:**
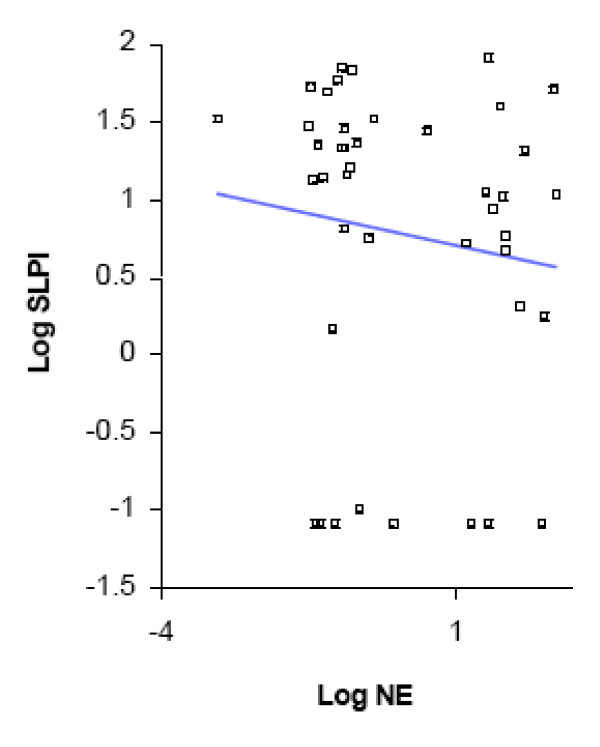
**Linear regression showing relationship of log-SLPI and log-NE levels for colonization samples only (R^2 ^= 0.02; p = 0.43)**.

We analyzed the data further to determine whether protease-antiprotease balance changes with colonization by *M. catarrhalis*, and to compare these changes with those seen with exacerbation. Colonization as well as exacerbation samples had significantly higher levels of NE and lower levels of SLPI, when compared to pre-acquisition samples (Table 3). The increase in NE from pre-acquisition levels was significantly higher in exacerbation as compared to the increase in colonization (p = 0.01, Fig. 4); while the decrease in SLPI from pre-acquisition levels was not significantly different between colonization and exacerbation (p = 0.7; Fig. 5). The relation between NE and SLPI, analyzed separately in exacerbation and colonization by linear regression, showed inverse relationships, but without reaching significance (Figures 6 and 7).

We conclude that acquisition of a new strain of *M. catarrhalis *is associated with worsening protease-antiprotease balance in favor of protease activity, and this occurs in colonization, albeit to a lesser extent than in exacerbations due to *M. catarrhalis*.

### Inter-relationship of airway inflammation and changes in NE

**Figure 8:**
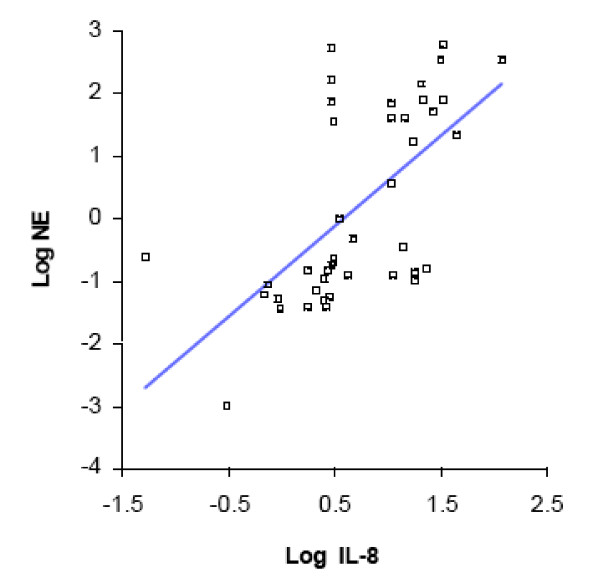
**Linear regression showing relationship of log-IL-8 and log-NE levels for colonization samples only (R^2 ^= 0.38; p =< 0.001)**.

**Figure 9:**
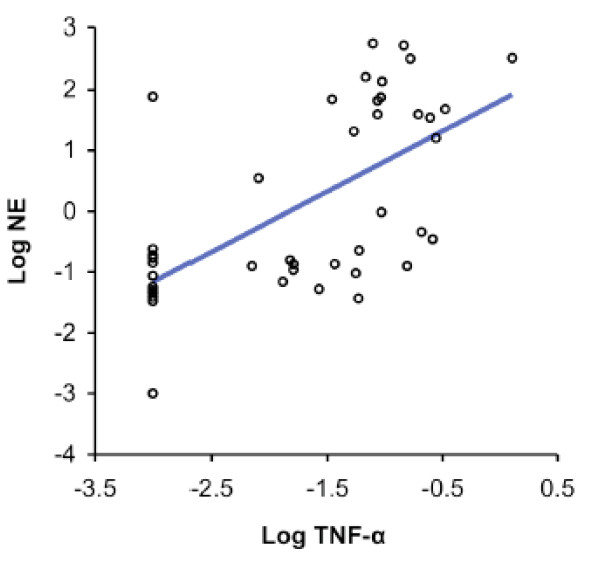
**Linear regression showing relationship of log-TNF-α and log-NE levels for colonization samples only (R^2 ^= 0.38; p =< 0.001)**.

A positive correlation between sputum levels of IL-8 and NE has been described in bacterial exacerbations of COPD [17]. In an effort to understand whether airway inflammation is related to protease-antiprotease balance in colonization by *M. catarrhalis*, we analyzed our data using linear regressions. This shows that sputum levels of IL-8 and TNF-α, which are markers of airway inflammation, correlate positively with levels of NE in colonization samples (Figs 8, 9). We conclude that airway inflammation in colonization due to *M. catarrhalis *correlates with changes in protease-antiprotease balance.

## Discussion

When COPD patients acquire a new strain of *M. catarrhalis*, approximately 50% suffer an acute exacerbation, and these exacerbation episodes are associated with a significant increase in neutrophilic airway inflammation from baseline [5,12]. The significance of colonization by *M. catarrhalis *in relation to airway inflammation in COPD and progression of disease was unclear. Though earlier cross-sectional studies had demonstrated that such colonization by bacterial pathogens, including *M. catarrhalis*, is pro-inflammatory, these studies lacked baseline values prior to acquisition of *M. catarrhalis *to determine accurately the change in inflammation associated with such colonization. The present study clearly demonstrates that colonization by *M. catarrhalis *is associated with enhanced airway inflammation, and that such inflammation is of lesser intensity than that seen in exacerbations associated with this pathogen. These findings imply that colonization by *M. catarrhalis *could contribute to tissue damage and disease progression in COPD.

Proteases play a major role in the disease progression in COPD, and any stimulus that enhances the proteolytic environment in the airways in COPD is likely to be detrimental and may contribute to disease progression. Previous studies have demonstrated that NE activity is increased during exacerbations of COPD, while SLPI level declines, thereby shifting the airway lumen environment towards proteolysis [8,17]. Our data extends these observations to colonization by *M. catarrhalis*, a finding that has important implications for progressive tissue damage in COPD.

The mechanisms underlying the reciprocal relationship between NE and SLPI are not fully understood, but there are several studies that suggest inactivation or degradation of SLPI by proteases. Studies with airway epithelial cell cultures show that exposure to NE increased mRNA expression for SLPI in these cells [20,21]. Immunohistochemistry demonstrated presence of SLPI in these cells [22]. However, in spite of increased production of SLPI induced by NE, cell supernatants in these experiments contained less SLPI compared to non-exposed cells [20,22,23]. This observation suggests extracellular destruction of SLPI. Several proteases, including NE, cathepsins and bacterial proteases can degrade SLPI in-vitro [24,25]. Another recently described potential mechanism of depletion of SLPI from respiratory secretions is adherence of SLPI-NE complexes to negatively charged membranes, including cell membranes [26]. It is not known whether *M. catarrhalis *has any direct down-regulating effect on SLPI production; this is an area for future investigation.

While we have considered airway inflammation and protease-antiprotease balance separately in results, it is highly likely that these are inter-related. IL-8 is a known chemo-attractant for neutrophils which produce NE [27,28]. Prior studies have shown that IL-8 levels in airway secretions correlate positively with NE levels in patients with chronic bronchitis during stable state and during exacerbations [4,8,17]. We extend these observations to colonization by M. catarrhalis as well.

Whether the changes we describe with colonization are specific to *M. catarrhalis *or present with other pathogenic bacteria as well is not clear at present. Many outer membrane components of *M. catarrhalis *trigger humoral immune responses [29,30] and probably are involved in triggering the inflammatory response in airways of COPD patients. *M. catarrhalis *activates NF-κB and MAPK pathways for increased production of inflammatory cytokines [31]. Moreover, *M catarrhalis *reduces the activity of histone deacetylase enzymes, promoting acetylation and easier access for the transcription factors like NF-kB to the genes for inflammatory cytokines like IL-8 [31]. Reduction of histone deacetylase activity is seen in COPD [32], suggesting that infection by bacteria such as *M. catarrhalis *could contribute to the dysregulated inflammation in COPD by this pathway. Additional studies to elucidate specific components of *M. catarrhalis *and pathways involved will lead to better understanding of bacteria-induced inflammation in COPD.

Limitations of this study are that sputum samples were not studied for neutrophil counts, viral and atypical bacterial pathogens, eosinophilic and other cellular inflammation. Neither did we examine indices of systemic inflammation in these episodes of *M catarrhalis *acquisition. We have examined only one protease present in human airways, though it is the major one (NE) and only one of the anti-proteases (SLPI). Other proteases such as matrix metalloproteases and anti-proteases such as a-1 antiprotease and elafin may play significant roles in colonization and exacerbation. Since our COPD clinic is based at a VA facility, almost all our patients are males. Our clinic recruits patients with smoking-induced chronic bronchitis with or without emphysema. Hence our findings may not be applicable to patients with pure emphysema alone. We have not examined long-term implications with regards to changes in lung function and functional status.

Co-existent viral infection could have contributed to the inflammation seen during colonization and exacerbation episodes with *M. catarrhalis*. However, we have earlier demonstrated that large increases in neutrophilic airway inflammation are characteristic of new bacterial strain acquisition [5]. Colonization episodes by definition were not associated with symptoms of respiratory infection, making a concomitant acute viral infection when these samples were collected unlikely. Though examining a single pathogen species in this study had several advantages, it does limit our ability to generalize these results to other bacterial pathogens in COPD.

We chose to study sputum rather than bronchoalveolar lavage (BAL) as repeated sampling using bronchoscopy would be invasive and paired pre-acquisition and acquisition samples would have been difficult to obtain. Though BAL cultures are more representative of lower airway bacteriology, we have shown in a prior study that sputum obtained with the protocol used in our study clinic approximates closely to induced sputum, and has the molecular markers of lower airway secretions [9].

## Conclusion

In conclusion, *M. catarrhalis *acquisition in COPD, with or without symptoms of exacerbation, is potentially detrimental to the course of COPD by enhancing airway inflammation and worsening protease-antiprotease imbalance.

## Abbreviations

**COPD**: Chronic obstructive pulmonary disease; **VA WNY**: Veterans Affairs Western New York Healthcare System; **IL-8**: Interleukin-8; **TNF-α**: Tumor necrosis factor-α; **NE**: Neutrophil elastase; **SLPI**: Secretory leukocyte protease inhibitor; **LTB-4**: Leukotriene B-4; **ELISA**: Enzyme linked immunosorbent assay **PWB**: Plate wash buffer; **TMB**: Tetramethyl benzidine; **DMSO**: Dimethyl sulfoxide **H**_2_**SO**_4_: Sulfuric acid.

## Competing interests

The authors declare that they have no competing interests.

## Authors' contributions

GIP and CTW performed the tests described. GIP analyzed data and drafted the manuscript. TFM and SS planned the project; reviewed and monitored its progress; helped in data analysis and edited the manuscript.

All authors read and approved the final manuscript.

## Pre-publication history

The pre-publication history for this paper can be accessed here:

http://www.biomedcentral.com/1471-2334/9/178/prepub
